# Topochemical Synthesis of Two‐Dimensional Transition‐Metal Phosphides Using Phosphorene Templates

**DOI:** 10.1002/anie.201911428

**Published:** 2019-11-18

**Authors:** Sheng Yang, Guangbo Chen, Antonio Gaetano Ricciardulli, Panpan Zhang, Zhen Zhang, Huanhuan Shi, Ji Ma, Jian Zhang, Paul W. M. Blom, Xinliang Feng

**Affiliations:** ^1^ Chair for Molecular Functional Materials Center for Advancing Electronics Dresden (cfaed) Technische Universität Dresden Mommsenstrasse 4 01062 Dresden Germany; ^2^ Max Planck Institute for Polymer Research Ackermannweg 10 55128 Mainz Germany

**Keywords:** black phosphorus, electrochemistry, topochemistry, transition-metal phosphides, two-dimensional materials

## Abstract

Transition‐metal phosphides (TMPs) have emerged as a fascinating class of narrow‐gap semiconductors and electrocatalysts. However, they are intrinsic nonlayered materials that cannot be delaminated into two‐dimensional (2D) sheets. Here, we demonstrate a general bottom‐up topochemical strategy to synthesize a series of 2D TMPs (e.g. Co_2_P, Ni_12_P_5_, and Co_*x*_Fe_2−x_P) by using phosphorene sheets as the phosphorus precursors and 2D templates. Notably, 2D Co_2_P is a p‐type semiconductor, with a hole mobility of 20.8 cm^2^ V^−1^ s^−1^ at 300 K in field‐effect transistors. It also behaves as a promising electrocatalyst for the oxygen evolution reaction (OER), thanks to the charge‐transport modulation and improved surface exposure. In particular, iron‐doped Co_2_P (i.e. Co_1.5_Fe_0.5_P) delivers a low overpotential of only 278 mV at a current density of 10 mA cm^−2^ that outperforms the commercial Ir/C benchmark (304 mV).

## Graphical abstract

**Material gains**: A general bottom‐up topochemical strategy has been developed to synthesize solution‐processable two‐dimensional transition‐metal phosphides, which exhibit semiconducting features and superior activity towards the oxygen evolution reaction. Notably, Co_2_P is a p‐type semiconductor, with a hole mobility of 20.8 cm^2^ V^−1^ s^−1^ at 300 K in field‐effect transistors.

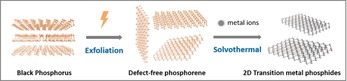

## Introduction

Transition‐metal phosphides (TMPs) display many interesting chemical and physical features, including superconductivity,^1^ thermoelectric properties,^2^ luminescence,^3^ and magnetism,^4^ derived from the unique role of the phosphorus atoms. The electronegativity of phosphorus is generally higher than those of metals, therefore, most TMPs are insulators or semiconductors, because their electron delocalization is strongly restricted.^5^ Recently, TMPs were re‐discovered as “star materials” in catalysis and energy harnessing. For example, cobalt‐ and iron‐based phosphides demonstrate high catalytic activity and long‐term stability for electrocatalytic water splitting in both acidic and basic solutions and are superior to many traditional catalysts based on expensive noble metals.^6^ From a fundamental point of view, the electrocatalytic behavior of TMPs is dominated by their interfacial chemistry as well as electronic structures, which govern, respectively, the formation of active surface sites and charge transport across the interfaces during the electrochemical reactions.^7^ Although the chemical compositions and crystal structures of TMPs are well known, experimental access to their electronic properties remains in a nascent stage, because TMPs are inherently nonlayered materials that cannot be delaminated into thin layers by top‐down exfoliation methods.^8^

Consequently, bottom‐up synthesis has become the mainstream approach to prepare multidimensional TMPs. Recent progress in this direction highlights the key impact of phosphorus (P) sources. Organophosphorus precursors, such as tri‐*n*‐octylphosphine (TOP)^9^ and other alkyl‐ and arylphosphines,^5^ are versatile P sources in wet‐chemical synthetic procedures. These result in a great number of monodispersed TMP nanoparticles at relatively low temperatures (220–385 °C) through a nucleation and growth mechanism.^10^ Alternatively, inorganic P sources, consisting of phosphates (e.g. (NH_4_)_2_HPO_4_),^11^ hypophosphines (e.g. NaH_2_PO_2_),^12^ tris(trimethylsilyl)phosphine (i.e. P(SiMe_3_)_3_),^13^ and red phosphorus,^14^ are widely used in solid–solid or gas–solid reactions. Nonetheless, these precursors require high‐temperature thermal annealing (e.g. 650 °C) to decompose or to transform into reactive intermediate species (e.g. PH_3_, white phosphorus gas),^15^ thus providing poor control over the shapes of the TMPs, which range from nanoparticles,^16^ bulk pieces,11a films,11b, 13 and nanorods12b to nanowire arrays.12a Although salt templating is able to guide the growth of TMP nanoplatelets,^17^ subsequent hydrogen (H_2_) annealing results in re‐formation of nanoscale clusters. To date, it remains a great challenge to synthesize 2D TMPs, especially, from conventional molecular precursors.

Herein, we demonstrate a novel topochemical strategy to prepare 2D TMPs in solution by using 2D phosphorene sheets as the P source and sacrificing templates. The intrinsic high chemical reactivity of phosphorene offsets the need for aggressive conditions^18^ and facilitates fast phosphidation of dispersed metal ions. The chemical structures of TMPs can be tailored by varying the feed species and ratios of metal salts, thereby resulting in 2D Co_2_P, Ni_12_P_5_, and Co_*x*_Fe_2−*x*_P nanosheets with mean lengths of 2.5 μm^2^ and thicknesses of 3.6 nm. Importantly, the 2D TMPs provide the feasibility to explore their charge‐transport properties as field‐effect transistors. Notably, the fabricated 2D Co_2_P shows p‐type semiconducting properties with a hole mobility of 20.8 cm^2^ V^−1^ s^−1^ at 300 K. When serving as electrocatalysts, Co_1.5_Fe_0.5_P sheets exhibit a low overpotential of 278 mV at a current density of 10 mA cm^−2^ and good stability (10 h) for the oxygen evolution reaction (OER), superior to the catalytic performance of commercial Ir/C. Our method opens up a new avenue for the design and construction of nonlayered 2D materials.

## Results and Discussion

**Figure 1:**
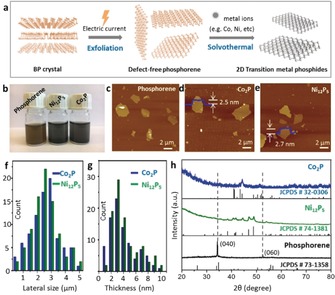
Synthesis of 2D TMPs. a) Exfoliation and transformation of bulk black phosphorus crystals into 2D TMPs. b) Optical images of phosphorene and 2D TMP dispersions in DMF (0.2 mg mL^*−*1^). c–e) AFM images of phosphorene, Co_2_P, and Ni_12_P_5_, respectively. f, g) Statistical analysis of Co_2_P and Ni_12_P_5_ flakes in terms of their dimensions and thickness. h) XRD patterns of thin films prepared by filtration of the dispersed 2D sheets.

Given that most of the TMPs are covalent compounds, in which the electron density easily shifts from the metal center (M) to the phosphorus atoms (P), the synthesis of TMPs does not require specific oxidation states of metal ions or phosphorus precursors.^5^ However, the formation of metal–metalloid (M‐P) bonds needs highly active precursors or harsh reaction conditions.^19^ In this respect, black phosphorus (BP) represents an ideal P source to realize the 2D structures of TMPs, because of its high reactivity arising from abundant lone pairs of electrons.^18^ Defect‐free phosphorene was prepared by electrochemical exfoliation of layered black phosphorus crystals by our previously reported method (Figure 1 a).^20^ Compared with other scalable exfoliation methods such as tip sonication^21^ and shear mixing,^22^ this mild intercalation strategy is able to maintain the structural integrity of phosphorene flakes. The metal sources consisted of several soluble coordination compounds, spanning from ammonium molybdate ((NH_4_)_2_MoO_4_), vanadyl acetylacetonate (VO(acac)_2_), ferrocene (Fe(C_5_H_5_)_2_), iron(III) acetylacetonate (Fe(acac)_3_), cobalt(II) acetylacetonate (Co(acac)_2_) and nickel(II) acetylacetonate (Ni(acac)_2_; see Scheme S1 in the Supporting Information). A solvothermal reaction was carried out between the mixed dispersion of exfoliated BP sheets, transition‐metal salt, and anhydrous *N*,*N*‐dimethylformamide (DMF) in an autoclave. We found that the Mo^VI^, V^IV^, and Fe^II^ salts failed to react with phosphorene, mostly because of competitive side reactions with the solvent or lattice mismatch with the target TMPs.^17^ The Fe^III^ salt mainly led to amorphous 2D Fe_2_P sheets (Figure S1), while the Co^II^ and Ni^II^ salts gave rise to 2D Co_2_P and Ni_12_P_5_ crystals, respectively (Figure 1 b). Note that, 2D Ni_2_P cannot be produced by this method as it is thermodynamically unstable.^23^ Dilute colloidal dispersions of the metal phosphide sheets (0.05 mg mL^−1^) show a clear Tyndall effect (Figure S2) and are stable for at least four weeks without sedimentation. In addition to the metal sources, the reaction temperature was also crucial. For example, at temperatures higher than 200 °C, the majority of the samples were metal oxide nanoparticles, which was ascribed to the strong reducing ability of DMF at high temperature.^24^ However, at 150 °C, TMPs were not formed even after 24 h. Therefore, a moderate temperature of 180 °C was selected as being optimal in terms of the resulting structures and reaction kinetics.

The morphology of the 2D sheets was examined by atomic force microscopy (AFM). As depicted in Figure 1 c, exfoliated BP flakes present mean diameters of (2.8±1.5) μm and thicknesses of (3.2±1.1) nm. This polydispersed size/thickness distribution is associated with a fragmentation‐exfoliation mechanism,^25^ well‐known for top‐down exfoliation methods. Based on energy‐dispersive X‐ray spectroscopy (EDX; Figures S3 and S4), the cobalt and nickel phosphides comprise atomic ratios of 2.1:1.0 (Co/P) and 2.3:1.0 (Ni/P), almost identical to their theoretical stoichiometry (i.e. 2.0:1.0 and 2.4:1.0, respectively). AFM images of both Co_2_P and Ni_12_P_5_ (Figure 1 d,e) display clean surfaces and well‐defined edges with height profiles of 2–3 nm. The transformation from phosphorene to metal phosphides did not cause apparent variation in the dimensions and thickness. For example, Co_2_P and Ni_12_P_5_ both had an average lateral size of 2.5 μm and thickness of 3.6 nm, close to the parameters of the parent phosphorene flakes, based on a statistical calculation of more than 80 flakes (Figure 1 f,g). By contrast, the crystal structures of the resulting TMP sheets were distinctly different. The starting phosphorene shows two strong characteristic (040) and (060) peaks at 34.2° and 52.4°, respectively, in the X‐ray diffraction (XRD) spectrum (Figure 1 h). However, neither peak was detectable after phosphidation reactions, thus indicating the broken ordering in the out‐of‐plane direction. Consequently, the XRD patterns of the products confirm the formation of orthorhombic Co_2_P (JCPDS No. 32‐0306) and tetragonal Ni_12_P_5_ (JCPDS No. 74‐1381). Co_2_P contains a tetrahedral and a pyramidal Co center, surrounded by four and five P atoms, respectively, while the P centers in Ni_12_P_5_ share 10 or 8 Ni atoms (Figure S5). Although the final products do not contain any phosphorene, the total yield is less than 100 %, because of the possible decomposition of phosphorene during the solvothermal process.

**Figure 2:**
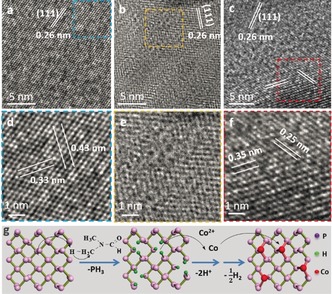
The topochemical synthetic process of 2D Co_2_P. a–c) High‐resolution TEM images of phosphorene sheets after carrying out reactions for 0 min, 20 min, and 60 min, respectively, and d–f) the corresponding magnified images. g) Proposed reaction mechanism.

Co_2_P was selected as a model material to track the structural evolution from phosphorene to 2D TMPs, and samples at different stages of the reactions were examined by high‐resolution transmission electron microscopy (HR‐TEM; Figure 2). Initially, phosphorene has the typical orthorhombic structure. The lattice fingers of 0.43 nm and 0.33 nm are in accordance with the respective [001] and [100] directions of the intact crystal (Figure 2 a,d).^26^ Each phosphorus atom connects to three adjacent phosphorus atoms through sp^3^ hybridization, thereby leading to two free electrons.^18^ As shown in Figure 2 b,e, lattice defects started to show up on the surface within 20 min and some phosphorus atoms were missing, while the intrinsic (111) facet of phosphorene remained on the main skeleton. Although a low density of defects did not cause a collapse of the 2D morphology, this intermediate geometry showed a clear distortion upon electron beam irradiation during the TEM measurement. As the solvothermal reaction proceeded, a new lattice orientation appeared on the phosphorene interfaces after 60 min, which corresponds to the incubation of Co_*x*_P (0<*x*<2; Figure 2 c,f). The spacing fingers of 0.35 nm and 0.25 nm were identified as the (001) and (210) facets of Co_2_P. Besides the Co_2_P lattice, the (111) facet of phosphorene was still pronounced. However, it vanished in the final Co_2_P product. This observation suggests that the synthesis of Co_2_P follows a conversion‐growth mechanism, in which cobalt atoms gradually react with phosphorus atoms and are incorporated into the 2D matrix.

To verify the underlying mechanisms, various combinations of precursors were tested in the same solvothermal environment. As an example, a dispersion of phosphorene in DMF became clear after 5 h reaction. The structural degradation of phosphorene indeed occurred, even though it was maintained in an oxygen‐free anhydrous system. In contrast, the solutions of metal salts in DMF resulted in amorphous solid or metal nitride (Figure S6). Thus, the interplay between the phosphorene, metal salts, and the solvent is essential to grow 2D TMPs. Despite DMF being a polar aprotic solvent, under suitable circumstances the DMF molecule loses a hydrogen atom and becomes a DMF radical, for example, by nucleophilic attack from a strong Lewis base (i.e. potassium methylate).^27^ Similarly, phosphorene can donate its unpaired electrons to DMF molecules and remove hydrogen atoms from their methyl groups. Therefore, it is rational to propose the following growth mechanisms (Figure 2 g):

At elevated temperature (e.g. 180 °C), the surrounding DMF molecules react at the edges and basal planes of phosphorene (denoted as BP), thereby causing phosphorus vacancies in the superlattice (i.e. BP_nVac_). Consequently, P atoms are detached in the form of PH_3_. Similar pathways towards a defective BP structure have been observed in the presence of water molecules at room temperature [Eq. (1)].^28^ The P atoms neighboring the vacancies continue to react with DMF molecules according to Equation (2). Afterwards, the growth of the 2D TMPs involves two steps:^29^ First, reduction of the metal ions to the metal (M) at hydrogenated P vacancies [Eq. (3)] followed by phosphidation of the metal clusters at the phosphorene interfaces [Eq. (4)].

(1) $$\mathrm{BP}\;+\;3\;n\;\left(\mathrm{CH}{}_{3}\right){}_{2}\mathrm{NCHO}\;\to \;\mathrm{BP}{}_{n\mathrm{Vac}}\;+\;n\;\mathrm{PH}{}_{3}\;+\;3\;n\;\mathrm{CH}{}_{3}\dot{C}H{}_{2}\mathrm{NCHO}:$$

(2) $$\mathrm{BP}{}_{n\mathrm{Vac}}\;+\;3\;n\;\left(\mathrm{CH}{}_{3}\right){}_{2}\mathrm{NCHO}\;\to \;\mathrm{BP}\text{-}H{}_{3n}\;+\;3\;n\;\mathrm{CH}{}_{3}\dot{C}H{}_{2}\mathrm{NCHO}:$$

(3) $$\mathrm{Co}{}^{2+}\;+\;\mathrm{BP}\text{-}H{}_{3n}\;\to \;\mathrm{Co}\;+\;\mathrm{BP}\text{-}H{}_{3n-2}\;+\;2\;H{}^{+}$$

(4) $$2\;n\;\mathrm{Co}\;+\;\mathrm{BP}\text{-}H{}_{3n-2}\;\to \;n\;\mathrm{Co}{}_{2}P\;+\;\frac{3n-2}{2}\;H{}_{2}$$

Despite the exact details of the phase transformation remaining elusive, the repetition of reactions (1)—(4) and subsequent structural rearrangement are responsible for transforming the entire phosphorene flakes into 2D metal phosphides.

Without metal ions in this system, the increasing density of P vacancies (BP_*n*Vac_ and BP‐H_3*n*_) was not able to support the 2D shape of phosphorene, which eventually broke down to soluble phosphorus‐containing compounds. It is worth noting that, as side reactions, nanoparticles could grow in the dispersion because of the accumulation of PH_3_. For example, with an extension of the reaction duration to 10 h, noticeable nanoparticles appeared on the surfaces of the TMP flakes (Figure S7). These experimental results agree well with our proposed mechanisms.

**Figure 3:**
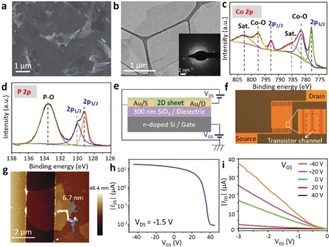
Structural and electronic properties of 2D Co_2_P. a) SEM images of 2D Co_2_P flakes on a Si substrate. b) TEM image of an individual Co_2_P flake (inset: SAED pattern). c, d) High‐resolution XPS spectra of Co 2p and P 2p, respectively. e) Schematic representation of the FET device in bottom‐contact bottom‐gate geometry. f) Optical image of the electrode‐patterned substrate. g) AFM image of a typical FET channel connected with a Co_2_P sheet between source and drain electrodes. h) Transfer curve of the FET device measured with a source‐drain bias of −1.5 V at 300 K. i) The *I*–*V* characteristics measured with various source–gate voltages.

Scanning electron microscopy (SEM) images (Figure 3 a, Figure S8a) confirm the polydispersed lateral dimensions of the Co_2_P and Ni_12_P_5_ flakes, which generally range from 1 to 5 μm, consistent with their AFM images. The representative TEM images (Figure 3 b, Figure S8b) display thin sheets with irregular shapes. The corresponding selected area electron diffraction (SAED) patterns indicate good crystallinity for both samples. The surface compositions and electron states were uncovered by X‐ray photoelectron spectroscopy (XPS; Figure 3 c,d). For Co_2_P, the characteristic peaks of Co‐P corresponding to Co 2p_3/2_ and Co 2p_1/2_ appear at 777.9 and 792.8 eV, respectively.^30^ Two satellite peaks at 785.6 and 802.5 eV and the peaks at 781.2 (2p_3/2_) and 797.1 eV (2p_1/2_) arise from Co−O bonding in oxidized Co species.^31^ Ni_12_P_5_ exhibits similar electron states. However, the peaks of Ni 2p_3/2_ and Ni 2p_1/2_ appear at higher binding energies of 852.8 and 870.1 eV, respectively (Figure S8d).^32^ The high‐resolution P 2p spectra (Figure 3 d, Figure S8e) of Co_2_P and Ni_12_P_5_ are very similar. The doublets at 129.3 and 130.2 eV are assigned to P−Co or P−Ni, while the broad peak at 133.3 eV originates from the oxidized P species.^30^ The slight surface oxidation is a common phenomenon of TMP nanomaterials as a result of air contact, and is consistent with many other reports.^6^, ^33^

The 2D TMP sheets constitute ideal platforms for the experimental studies on their electronic properties in field‐effect transistor (FET) devices. Bottom‐gate and gold bottom‐contact FET devices were fabricated by spin coating of dilute metal phosphide dispersions onto commercial electrode‐patterned substrates with 300 nm SiO_2_ dielectric layers (Figure 3 e,f), followed by thermal annealing in vacuum to remove the residual solvents and surface oxide layers. All measurements on the charge‐transport behavior were recorded at room temperature (300 K) in vacuum (ca. 1×10^−5^ mbar). As shown in Figure 3 g, a thin Co_2_P layer (6.7 nm thick) bridges the source and drain electrodes with a channel width of 2.5 μm. The transfer curve (Figure 3 h) proves that the synthesized 2D Co_2_P sheet is a p‐type semiconductor with a hole mobility of 20.8 cm^2^ V^−1^ s^−1^, calculated from the linear slope of the source‐drain current (*I*_ds_) plot. The mobility in 2D Co_2_P is on the same order of magnitude as few‐layer metal chalcogenides, varying from 10 to 50 cm^2^ V^−1^ s^−1^ at 300 K.^34^ The *I*–*V* characteristics show good linearity in the regime of the source‐drain voltage (*V*_ds_) from −3 V to 0 V (Figure 3 i). The *I*_on_/*I*_off_ ratio based on drain‐current modulation is 1.8×10^3^. Our observation agrees with early experimental studies that cobalt phosphides have narrow band gaps of 0.67–0.88 eV in mixed phases,^35^ although some density of states (DOS) calculations claim that the Co_2_P structure is metallic without a band gap.^36^ In comparison, the 2D Ni_12_P_5_ sheet demonstrates similar features but has a lower hole mobility of 8.7 cm^2^ V^−1^ s^−1^ and an *I*_on_/*I*_off_ ratio of 1.6×10^2^ (Figure S9). Ultrathin 2D TMPs are promising semiconductor electrocatalysts despite their intrinsic low carrier concentration.^2^ At the semiconductor–electrolyte interfaces, the pronounced “self‐gating” phenomenon^37^ switches the surface conductance between “on” and “off” states, which correspond to highly conductive and insulating phases, respectively. The charge‐transfer process occurs primarily at the active regions, while carrier concentration accumulates to an extremely high level (over 10^14^ e cm^−2^) at inert regions.^37^ The capability for surface charge modulation strongly supports their fast charge transport kinetics in the electrocatalytic reactions.

**Figure 4:**
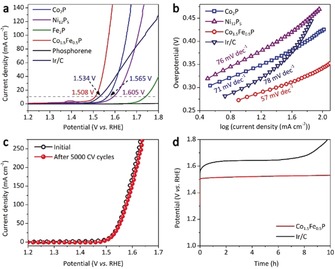
Electrocatalytic performances of 2D TMPs towards the OER. a) OER polarization curves of the Co_2_P, Ni_12_P_5_, Fe_2_P, Co_1.5_Fe_0.5_P, phosphorene, and Ir/C electrocatalysts. b) Corresponding Tafel plots of Co_2_P, Ni_12_P_5_, Co_1.5_Fe_0.5_P, and Ir/C. c) Polarization curves of the 2D Co_1.5_Fe_0.5_P before and after 5000 CV cycles. d) Long‐term OER stability test of the Co_1.5_Fe_0.5_P and Ir/C at a current density of 10 mA cm^−2^.

As a proof of concept, 2D Co_2_P, Ni_12_P_5_, and Fe_2_P were prepared to illustrate their potential applications in the OER. A typical three‐electrode system in a N_2_‐saturated 1.0 m KOH aqueous electrolyte was applied, using an Ag/AgCl electrode and a Pt wire as the reference and counter electrodes, respectively. All potentials were referred to the reversible hydrogen electrode (RHE), and the ohmic potential drop from the electrolyte resistance were subtracted. As depicted in Figure 4 a, phosphorene and Fe_2_P nanosheets have sluggish activity, whereas Co_2_P and Ni_12_P_5_ exhibit smaller onset OER overpotentials of 280 mV and 285 mV, respectively. Metal doping or alloying is an efficient method to reduce the kinetic energy barriers, and thus to improve the electrochemical performance of TMPs.^38^ As expected, iron‐doped bimetallic phosphide Co_*x*_Fe_2−*x*_P (0<*x*<2) shows enhanced electrocatalytic activity (Figures S10 and S11). Remarkably, when *x*=1.5, Co_1.5_Fe_0.5_P generates oxygen at an overpotential of only about 180 mV, which is substantially lower than that of commercial Ir/C (ca. 260 mV; Figure S12). At a current density of 10 mA cm^−2^, Co_1.5_Fe_0.5_P delivers a low overpotential of 278 mV, superior to those of Co_2_P (335 mV), Ni_12_P_5_ (375 mV), and Ir/C catalysts (304 mV). The catalytic performance of Co_1.5_Fe_0.5_P also outperforms many state‐of‐the‐art OER electrocatalysts, such as CoMnP nanoparticles,^39^ NiCoP/C nanoboxes,^40^ Mn‐Co oxyphosphide multishelled particles,^41^ CoFe‐layered double hydroxides,^42^ meso/micro‐FeCoN_*x*_‐CN,^43^ and double perovskite LaFe_*x*_Ni_1−*x*_O_3_ nanorods^44^ that have overpotentials ranging from 290 to 450 mV (Table S1). Moreover, Co_1.5_Fe_0.5_P undergoes a rapid charge‐transfer process, implied by its electrochemical impedance (Figure S13). The Tafel slope of Co_1.5_Fe_0.5_P (57 mV decade^−1^) is lower than that of Co_2_P (71 mV decade^−1^), Ni_12_P_5_ (76 mV decade^−1^), and Ir/C (78 mV decade^−1^), thus suggesting it has fast OER kinetics (Figure 4 b). The overpotential of Co_1.5_Fe_0.5_P slightly increases by only 1 mV after 5000 cyclic voltammetry (CV) scans performed between 1.0 and 1.6 V at a scan rate of 50 mV s^−1^ (Figure 4 c), thus indicating its excellent electrocatalytic durability. According to a long‐term electrocatalytic OER process at a constant current density of 10 mA cm^−2^, Co_1.5_Fe_0.5_P shows excellent stability that retains a steady OER activity over a period of 10 h. By contrast, the Ir/C catalyst gradually loses its activity during the measurements (Figure 4 d). The remarkable electrocatalytic performances of 2D Co_1.5_Fe_0.5_P are correlated with three major factors: 1) Intrinsic high catalytic activities of Co and Fe as well as the highly exposed surfaces permit rapid oxidation and transformation into the corresponding active metal oxide/oxyhydroxides;^45^ 2) the “self‐gating” effect largely improves the concentration of the surface charge during the OER;^37^ and 3) the confined 2D sheet structure ensures fast charge transport at the interfaces.

## Conclusion

In summary, we have developed a versatile topochemical strategy to prepare various solution‐processable 2D metal phosphides. Transition‐metal precursors, including cobalt and nickel salts, are able to transform phosphorene nanosheet templates into the corresponding 2D TMPs with an average lateral size of 2.5 μm and thickness of 3.6 nm. Field‐effect transistors disclose the underlying p‐type semiconducting properties of as‐prepared 2D TMPs, in which, 2D Co_2_P demonstrates a hole mobility of 20.8 cm^2^ V^−1^ s^−1^ at room temperature. The unique electronic structure and highly exposed active surface sites enable high electrocatalytic activity of 2D TMPs for the OER. Remarkably, iron‐doped Co_2_P (i.e. Co_1.5_Fe_0.5_P) exhibits a low overpotential of 278 mV at a current density of 10 mA cm^−2^ and superior stability over a period of 10 h without apparent performance decay. This topochemical method opens up new horizons for the design and synthesis of nonlayered 2D materials, which do not fit traditional exfoliation approaches.

## Conflict of interest

The authors declare no conflict of interest.

## Supporting information

As a service to our authors and readers, this journal provides supporting information supplied by the authors. Such materials are peer reviewed and may be re‐organized for online delivery, but are not copy‐edited or typeset. Technical support issues arising from supporting information (other than missing files) should be addressed to the authors.

Supplementary
