# Supplementary material for: Topochemical Synthesis of Two‐Dimensional Transition‐Metal Phosphides Using Phosphorene Templates
Source: Angew Chem Int Ed Engl. 2019 Nov 18;59(1):465–70. doi: 10.1002/anie.201911428 (PMC6972539; doi:10.1002/anie.201911428)
Supplement: Supplementary file 1 — Supplementary [file ANIE-59-465-s001.pdf]

Angewandte Chemie
Eine Zeitschrift der Gesellschaft Deutscher Chemiker

Supporting Information

Topochemical Synthesis of Two-Dimensional Transition-Metal Phosphides Using Phosphorene Templates

*Sheng Yang[+], Guangbo Chen[+], Antonio Gaetano Ricciardulli[+], Panpan Zhang[+], Zhen Zhang, Huanhuan Shi, Ji Ma, Jian Zhang, Paul W. M. Blom, and Xinliang Feng**

anie_201911428_sm_miscellaneous_information.pdf

Experimental

Exfoliation of Black Phosphorus (BP). Bulk BP crystal was exfoliated according to our previous report using an electrochemical approach.[1] In brief, a small piece of BP crystal (50 mg) was placed as cathode in a two-electrode cell, which included a platinum anode and anhydrous organic electrolyte (25 mL). The electrolyte was made of 0.1 M tetra-*n*-butyl-ammonium bisulfate (TBA HSO_4) in deoxygenated propylene carbonate (PC). A stable working bias (–8.0 V) was applied between cathode and anode to trigger the exfoliation. To avoid possible degradation of exfoliated phosphorene flakes, the entire exfoliation process (30 min) was kept in an argon-filled glove box. Afterwards, the exfoliated flakes were carefully rinsed with anhydrous isopropyl alcohol (IPA) to clean the residual electrolyte, then dispersed into anhydrous *N,N*-dimethylformamide (DMF) by mild sonication in the ice bath (15 min). The as-prepared dispersion was centrifuged at 2000 rpm, 10 min to discard the suspended thick flakes and un-exfoliated large pieces. The brown supernatant (ca. 0.60 mg mL^{-1}) was collected for further experiments.

Synthesis of 2D Transition Metal Phosphides (TMPs). Cobalt (II) acetylacetonate (30 mmol) or Nickel (II) acetylacetonate (30 mmol) was mixed with the DMF dispersion of phosphorene (0.60 mg mL^{-1}, 15 mL) under constant stirring (400 rpm, 20 min). The homogeneous mixture was transferred into a 30 mL Teflon lined autoclave. Note that, these steps were prepared carefully inside glove box to prevent the phosphorene from oxidation. Then, the whole setup was tightly sealed and transferred into a heating oven, kept at 180 ^{o}C for 5 h under air circulation. When the reaction completed, the color of suspension turned from brown to dark gray. The resulted TMP flakes were centrifuged (2000 rpm, 10 min), washed at least three times by anhydrous IPA and re-dispersed in fresh DMF. To prepare bimetallic metal phosphides ($Co_xFe_{2-x}P$, $0<x<2$), the experimental parameters were the same except the feeding ratios of Cobalt (II) acetylacetonate and Iron (III) acetylacetonate, which varied from 1:1 (15:15 mmol), 2:1 (20:10 mmol), 3:1 (22.5:7.5 mmol), 4:1 (24:6 mmol), to 5:1(25:5 mmol).

Instrumentation. The morphology of phosphorene and TMP flakes were studied by scanning electrode microscope (SEM, Zeiss Gemini 500), transmission electron microscopy (TEM, Zeiss Libra 120 kV). The thickness of 2D flakes was checked by Bruker Multimode 8 atomic force microscope (AFM). XPS analyses were carried out using an AXIS Ultra DLD X-ray photoelectron spectrometer with a basic chamber pressure of 10^{-10} mbar and with an Al anode as the X-ray source (x-ray radiation of 1486.7 eV). X-ray diffraction (XRD) was performed on a PW1820 powder diffrationmeter (Phillips) with Ni-filtered Cu Kα radiation (1.5406 Å) at a scan rate of 0.02 deg. s^{-1}. The electrocatalytic measurements were carried out with WaveDriver 20 (Pine Research Instrumentation) and CHI 660E Potentiostat system (CH Instruments).

Field Effect Transistors. FET devices were fabricated in an argon-filled glove box (O_2<0.1 ppm, H_2O<0.1 ppm) using heavily n-doped silicon substrates that were covered with a 300 nm-thick thermally grown oxide dielectric. Bottom-gate and bottom-contact devices were prepared by spin-coating (2000 rpm, 30 s) of the 2D TMP dispersion in isopropanol. The concentration of 2D Co_2P and $Ni_{12}P_5$ dispersion was 0.10 mg mL^{-1}. Afterwards, the devices were annealed at 300 °C overnight in vacuum to remove any residual solvents and oxide layers on the surfaces. The hole mobility was calculated from the linear regime of the transfer curves based on the following equation:

$$\mu = \frac{L}{WC_iV_d} \times \frac{\Delta I_d}{\Delta V_g}$$

(where C_i is the dielectric capacitance (11 nF cm^{-2}), L and W are the channel length and width between source/drain electrode, respectively).

All device tests were carried out under vacuum using a Hewlett Packard 4155B semiconductor parameter analyzer.

Electrochemical Measurements. The oxygen evolution reaction (OER) activity was evaluated in a three-electrode configuration using a rotating disk electrode (RDE) (PINE Research Instrumentation) at a rotation speed of 1600 rpm. All the measurements were performed at ambient condition in a N_2-saturated 1.0 M KOH alkaline solution. A standard Ag/AgCl electrode and a platinum wire were used as the reference and counter electrode,

respectively. A glassy carbon (GC) disk electrode (5 mm in diameter) was used as the working electrode. Benchmark Ir/C (20 %) was purchased from FuelCellStore.The catalyst suspension was prepared by dispersing 2 mg of catalyst in 0.4 mL of solution containing 0.38 mL of ethanol and 20 μL of 5 wt.% Nafion solution followed by ultrasonication for 30 min. Then, 10 μL of the above suspension was dropped on the polished GC electrode and then dried at room temperature. Potentials in this work were referred to the reversible hydrogen electrode (RHE) by calibrating in H_2-saturated 1.0 M aqueous solution.

All potentials were corrected to eliminate electrolyte resistances unless special noted. Electrochemical impedance spectroscopy (EIS) measurements were performed at 1.51 V (*vs.* RHE) with frequency from 0.01 Hz to 100 kHz at an alternating current voltage amplitude of 10 mV. The electrochemically active surface areas (ECSA) were studied based on the electrochemical double-layer capacitance of various electrocatalysts at non-faradaic potentials. By plotting the difference of current density (J) between the anodic and cathodic sweeps ($J_{anodic} - J_{cathodic}$) at 1.35 V (*vs.* RHE) against the scan rate, a linear trend was observed. The slope of the fitting line is equal to twice the geometric double layer capacitance (C_{dl}). Namely, $C_{dl} = (J_{anodic} - J_{cathodic})/2\nu$.

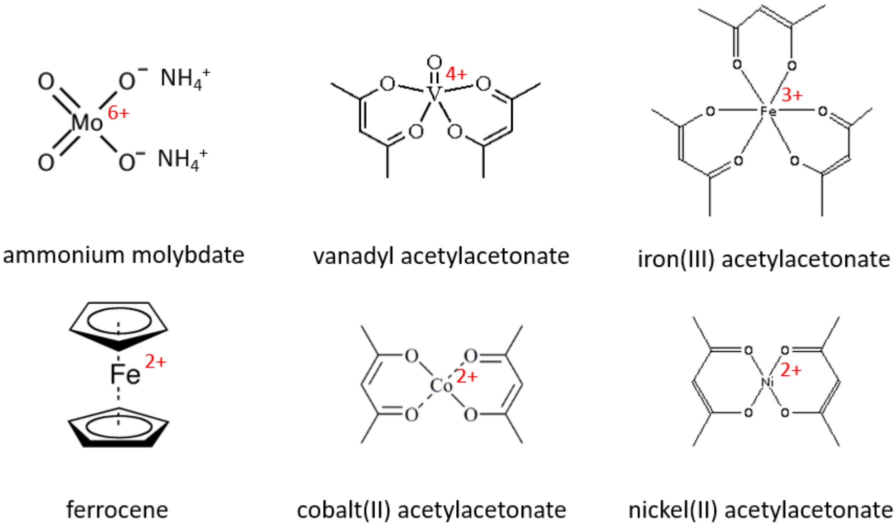

Scheme S1. Chemical structures of the transition metal sources used in this work

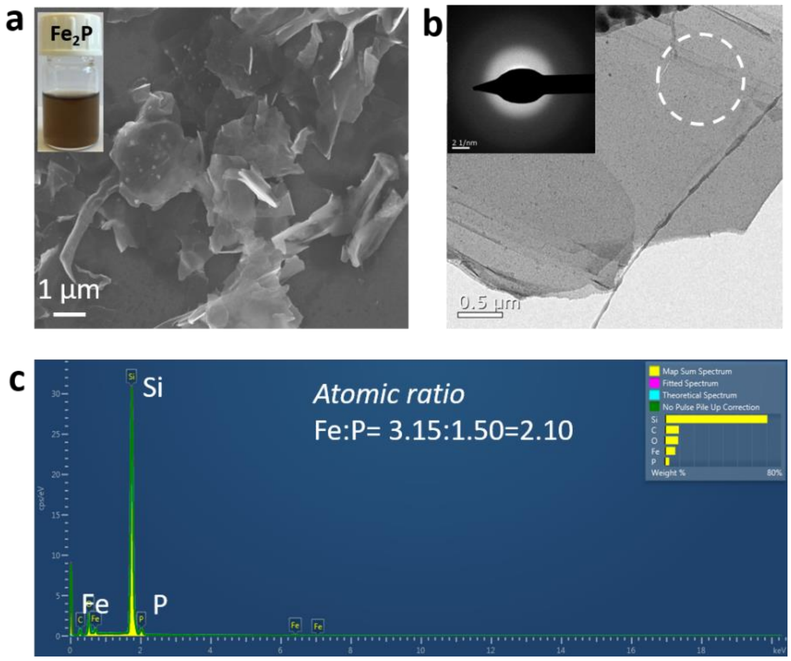

Figure S1. Structural characterization of amorphous Fe_2P sheets. a) SEM image of Fe_2P sheets on Si substrate (Inset: optical picture of dispersion in DMF), b) TEM image of two overlapped sheets (Inset: the diffraction pattern on a selected area confirms an amorphous structure), c) The atomic ratio of iron and phosphorus from elemental mapping is 2.1:1.

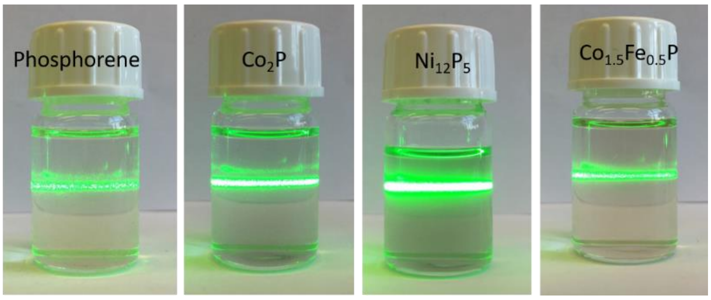

Figure S2. Tyndall effect of colloidal dispersions of phosphorene and 2D TMPs in DMF with a concentration of 0.05 mg mL^{-1}.

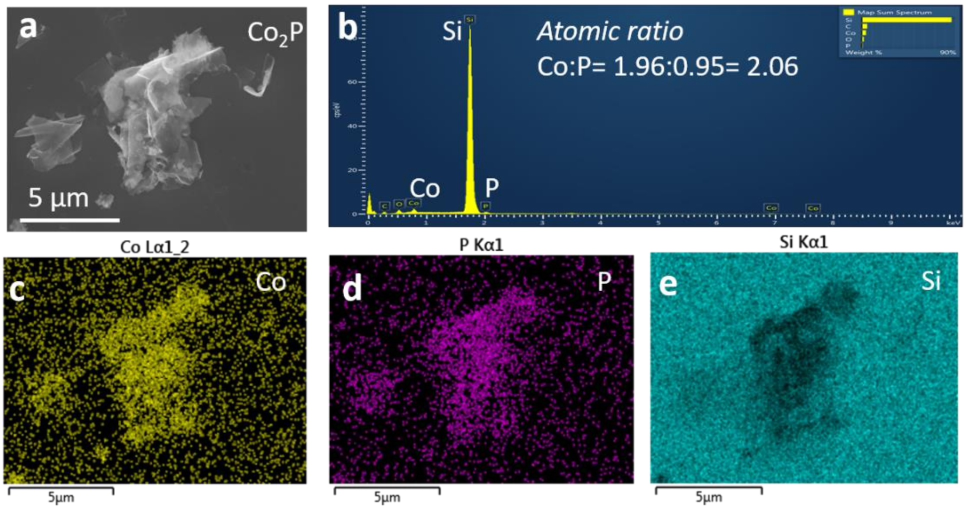

Figure S3. Elemental mapping of Co_2P sheets on Si substrate. a) SEM image of selected Co_2P sheets. b-e) Elemental distribution from EDX mapping. Cobalt and phosphorus atoms are homogeneous dispersed in the entire sheets.

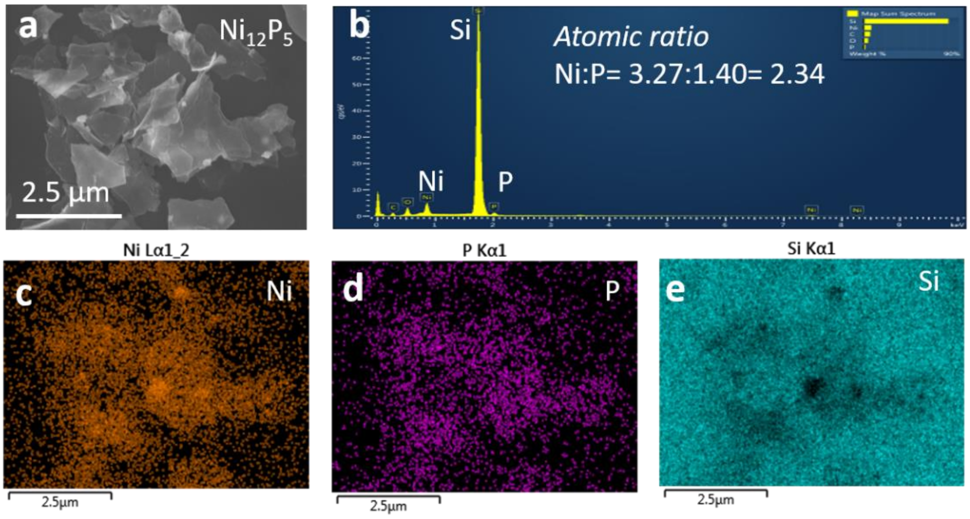

Figure S4. Elemental mapping of $Ni_{12}P_5$ sheets on Si substrate. a) SEM image of selected $Ni_{12}P_5$ sheets. b-e) Elemental distribution from EDX mapping.

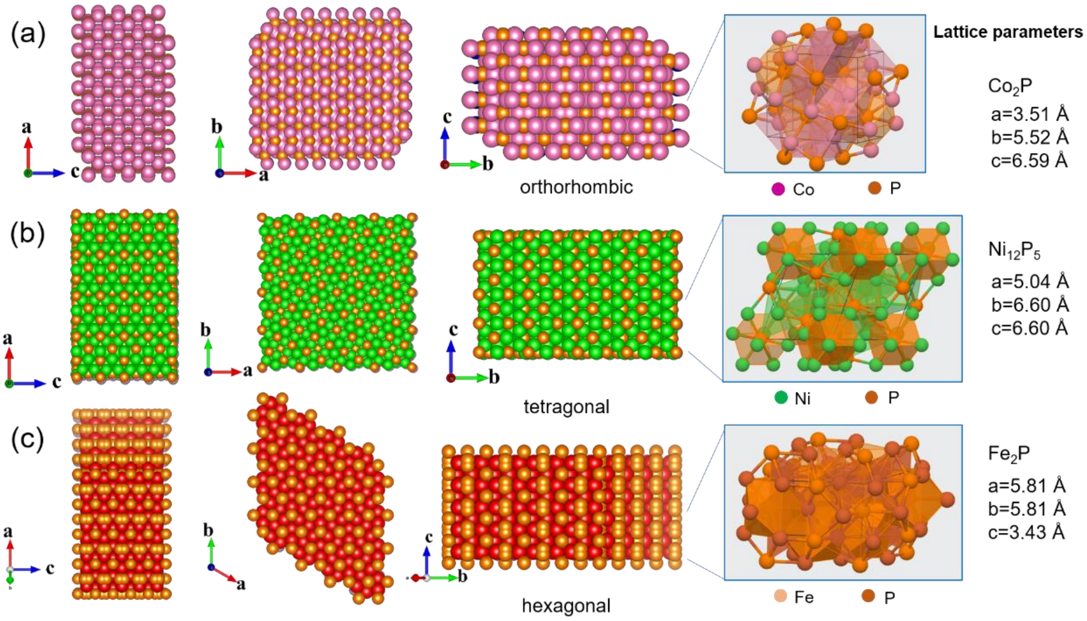

Figure S5. Crystal structures of two-dimensional a) orthorhombic Co_2P, b) tetragonal $Ni_{12}P_5$ and c) hexagonal Fe_2P. (The single-unit structures were generated from an online crystal database https://materialsproject.org, access date 30 Sep 2019).

The lattice parameters of Co_2P and $Ni_{12}P_5$ along the c-axis are very close to the monolayer thickness of phosphorene (0.5-0.7 nm).[2] Therefore, the expected thickness of 2D TMPs is the same as their phosphorene precursors.

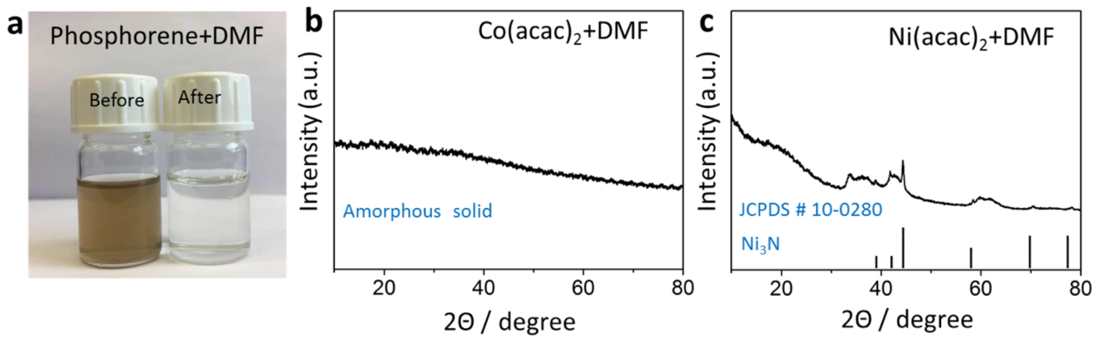

Figure S6. a) Optical images of phosphorene dispersion in DMF before (left) and after (right) solvothermal treatment; XRD spectra of the solids obtained from the reactions between b) cobalt salt and DMF, c) nickel salt and DMF.

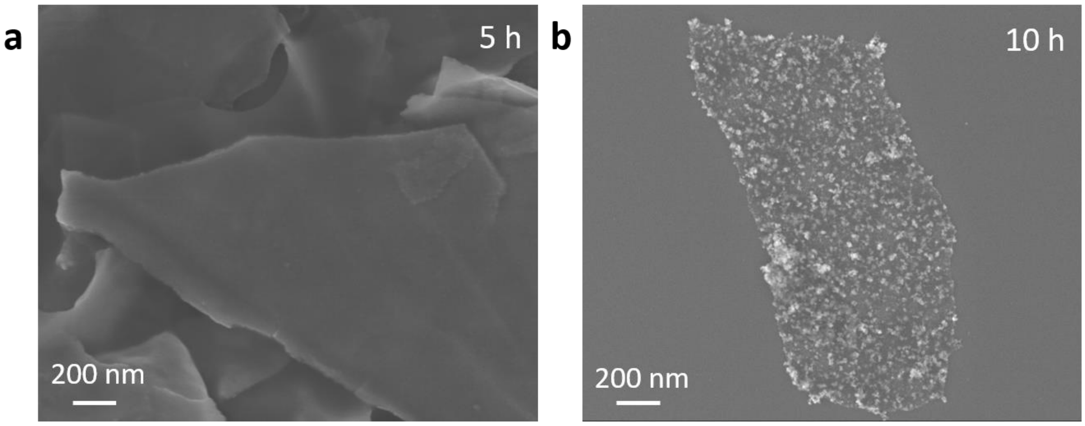

Figure S7. SEM images of Co_2P sheets after reaction for a) 5 hours and b) 10 hours. Nanoparticles start to grow on sheets and become apparent after prolonged reaction, due to side reactions in solution.

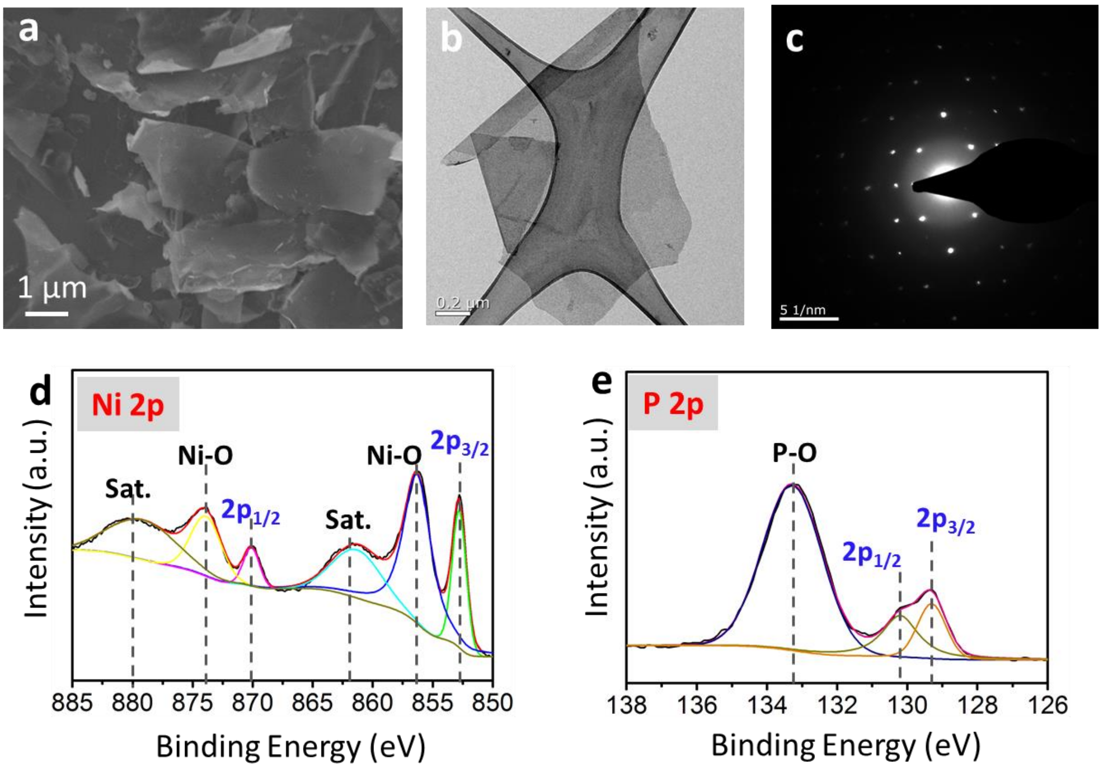

Figure S8. a) SEM images of $Ni_{12}P_5$ flakes on Si substrate. b) TEM image of a single $Ni_{12}P_5$ flake and c) its corresponding SAED pattern. d,e) High-resolution XPS spectrum of Ni 2p and P 2p, respectively.

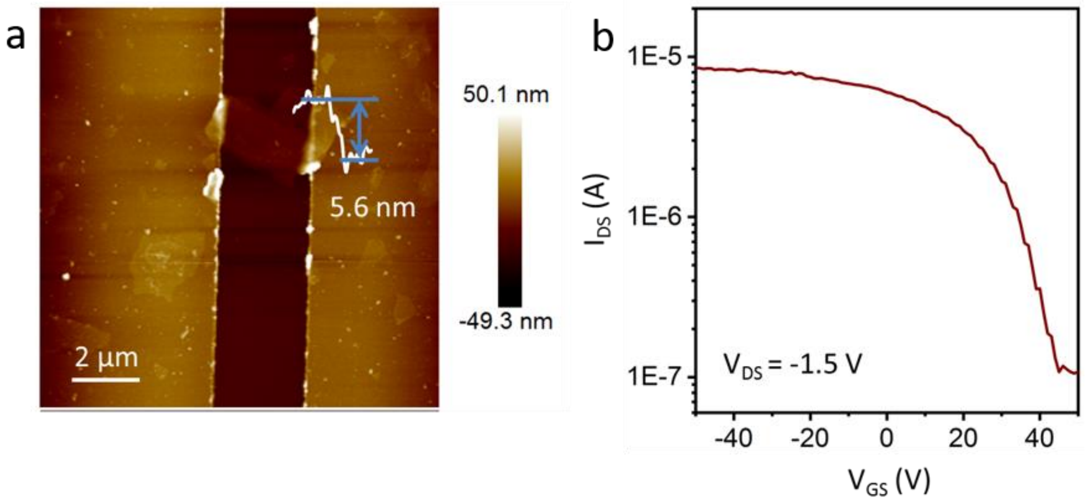

Figure S9. a) AFM image of a typical FET device with a $Ni_{12}P_5$ sheet (5.6 nm thick) bridging source and drain electrodes. b) Transfer curve of the device measured with a V_{DS} of -1.5 V.

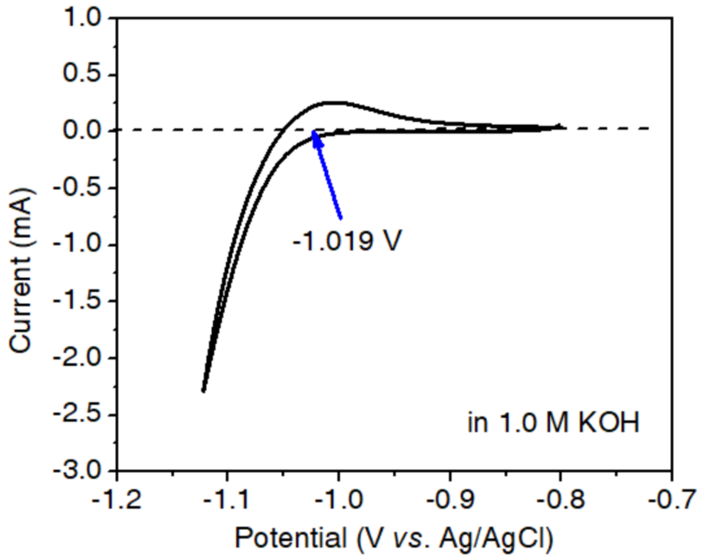

Figure S10. Potential calibration of the Ag/AgCl reference electrode in 1.0 M KOH aqueous solution.

The Ag/AgCl electrode was calibrated with respect to the reversible hydrogen electrode (RHE) in H_2 saturated 1.0 M KOH solution. Cyclic voltammetry (CV) curve was measured at a scan rate of 1 mV s^{-1}, and thermodynamic potential for the hydrogen electrode reactions was selected based on the average value of scanning potentials at I=0 mA.

E (RHE) = E (Ag/AgCl) + 1.019 V.

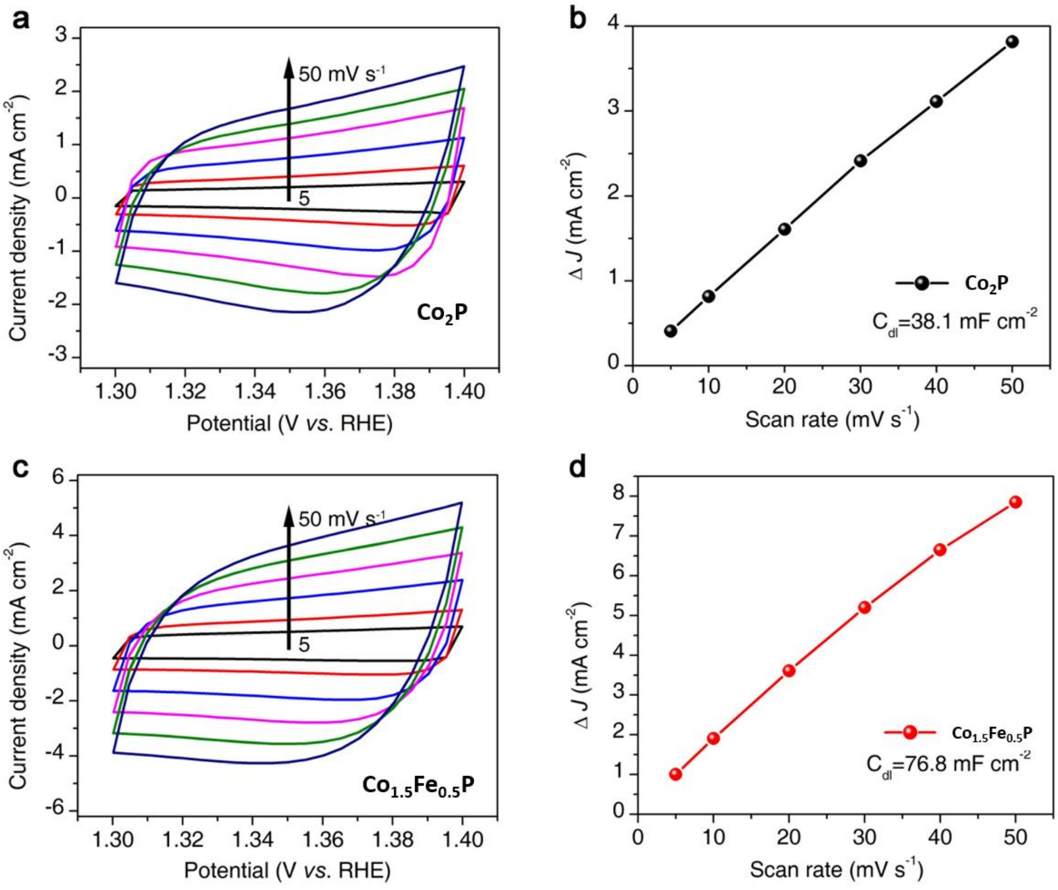

Figure S11. CV curves of a) Co_2P, c) $Co_{1.5}Fe_{0.5}P$ modified electrodes in the double layer region at scan rates of 5, 10, 20, 30, 40 and 50 mV s^{-1} in 1.0 M KOH. The differences in the current densities (J_{anodic} - $J_{cathodic}$) plotted against the scan rates, b) Co_2P and d) $Co_{1.5}Fe_{0.5}P$. The slope of the fitting line is equal to the geometric double layer capacitance (C_{dl}), which is proportional to the effective electrode surface area of the materials.

The electrochemical double-layer capacitances show that the $Co_{1.5}Fe_{0.5}P$ (76.8 mF cm^{-2}) has a larger active surface area than the Co_2P (38.1 mF cm^{-2}).

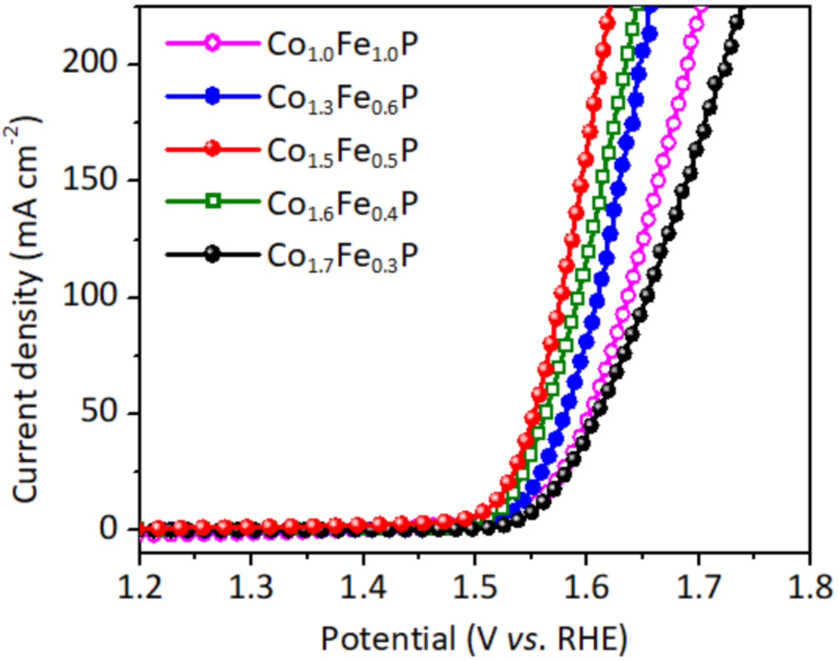

Figure S12. OER polarization curves of $Co_{2-x}Fe_xP$ electrocatalysts with different Co:Fe molar ratios of 1:1, 2:1, 3:1, 4:1, 5:1.

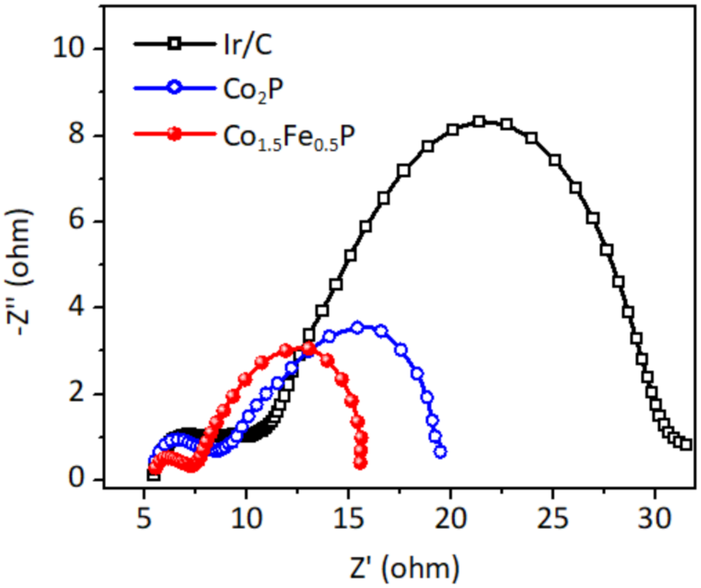

Figure S13. Electrochemical impendence spectra (EIS) of Co_2P, $Co_{1.5}Fe_{0.5}P$ and Ir/C benchmark.

EIS measurements were conducted in Ar-saturated 1.0 M KOH aqueous solution at 1.51 V *vs.* RHE with 10 mV AC potential from 10 kHz to 0.01 Hz. The measured impedances presented in the form of imaginary (*Im*) *vs*. real (*Re*) parts at various frequencies. The high frequency interception of the Re-axis represents the resistance of the electrodes and the width of the semicircle on the Re-axis corresponds to the charge-transfer resistances, indicating the overall kinetic effects. Clearly, three electrocatalysts exhibited similar intrinsic resistance, while the charge-transfer resistance of the $Co_{1.5}Fe_{0.5}P$ was much lower than that of the Ir/C and Co_2P, suggesting a faster OER kinetic process on the $Co_{1.5}Fe_{0.5}P$ electrocatalyst.

Table S1. Brief comparison of the electrocatalytic OER performance of the $Co_{1.5}Fe_{0.5}P$ with the reported electrocatalysts in 1.0 M KOH solution.

Catalysts	Overpotential at 10 mA cm^{-2} (mV)	Tafel slope (mV $decade^{-1}$)	Reference
Ni_2P nanowires	290	47	[3]
CoP hollow Polyhedron	400	57	[4]
MnCoP nanoparticles	330	61	[5]
O-Ni_2P nanosheets	347	63	[6]
Mn-Co oxyphosphide multi-shelled particles	320	52	[7]
NiCoP/C nanoboxes	330	96	[8]
Co_xP	399	214.3	[9]
Ag@Co_xP	310	76.4	[9]
Co_2P/BP	380	78	[10]
CoP nanosheets	315	80	[11]
$Fe_{1.1}Mn_{0.9}P$ nanorods	440	39	[12]
Co/BP nanosheets	310	61	[13]
CoP/rGO	340	66	[14]
γ-CoOOH nanosheets	300	38	[15]
CoFe-LDHs	321	57	[16]
Co_3O_4/Co-Fe oxide	297	61	[17]
Cobalt Phyllosilicate	364	60	[18]
Co–C_3N_4/CNT	380	68.4	[19]
Meso/micro-$FeCoN_x$-CN	450	57	[20]
2D Co_2P	335	71	This work
2D $Co_{1.5}Fe_{0.5}P$	278	57	This work

Reference

[1] S. Yang, K. Zhang, A. G. Ricciardulli, P. Zhang, Z. Liao, M. R. Lohe, E. Zschech, P. W. M. Blom, W. Pisula, K. Müllen, X. Feng, *Angew. Chem. Int. Ed.* **2018**, *57*, 4677-4681.

[2] D. Hanlon, C. Backes, E. Doherty, C. S. Cucinotta, N. C. Berner, C. Boland, K. Lee, A. Harvey, P. Lynch, Z. Gholamvand, S. Zhang, K. Wang, G. Moynihan, A. Pokle, Q. M. Ramasse, N. McEvoy, W. J. Blau, J. Wang, G. Abellan, F. Hauke, A. Hirsch, S. Sanvito, D. D. O'Regan, G. S. Duesberg, V. Nicolosi, J. N. Coleman, *Nat. Commun.* **2015**, *6*, 8563.

[3] L.-A. Stern, L. Feng, F. Song, X. Hu, *Energy Environ. Sci.* **2015**, *8*, 2347-2351.

[4] M. Liu, J. Li, *ACS Appl. Mater. Interfaces* **2016**, *8*, 2158-2165.

[5] D. Li, H. Baydoun, C. N. Verani, S. L. Brock, *J. Am. Chem. Soc.* **2016**, *138*, 4006-4009.

[6] Z. Li, X. Dou, Y. Zhao, C. Wu, *Inorg. Chem. Front.* **2016**, *3*, 1021-1027.

[7] B. Y. Guan, L. Yu, X. W. Lou, *Angew. Chem. Int. Ed.* **2017**, *56*, 2386-2389.

[8] P. He, X.-Y. Yu, X. W. Lou, *Angew. Chem. Int. Ed.* **2017**, *56*, 3897-3900.

[9] Y. Hou, Y. Liu, R. Gao, Q. Li, H. Guo, A. Goswami, R. Zboril, M. B. Gawande, X. Zou, *ACS Catal.* **2017**, *7*, 7038-7042.

[10] J. Wang, D. Liu, H. Huang, N. Yang, B. Yu, M. Wen, X. Wang, P. K. Chu, X.-F. Yu, *Angew. Chem. Int. Ed.* **2018**, *57*, 2600-2604.

[11] X. Xiao, C.-T. He, S. Zhao, J. Li, W. Lin, Z. Yuan, Q. Zhang, S. Wang, L. Dai, D. Yu, *Energy Environ. Sci.* **2017**, *10*, 893-899.

[12] D. Li, H. Baydoun, B. Kulikowski, S. L. Brock, *Chem. Mater.* **2017**, *29*, 3048-3054.

[13] F. Shi, Z. Geng, K. Huang, Q. Liang, Y. Zhang, Y. Sun, J. Cao, S. Feng, *Adv. Sci.* **2018**, *5*, 1800575.

[14] L. Jiao, Y.-X. Zhou, H.-L. Jiang, *Chem. Sci.* **2016**, *7*, 1690-1695.

[15] J. Huang, J. Chen, T. Yao, J. He, S. Jiang, Z. Sun, Q. Liu, W. Cheng, F. Hu, Y. Jiang, Z. Pan, S. Wei, *Angew. Chem. Int. Ed.* **2015**, *54*, 8722-8727.

[16] Y. Wang, Y. Zhang, Z. Liu, C. Xie, S. Feng, D. Liu, M. Shao, S. Wang, *Angew. Chem. Int. Ed.* **2017**, *56*, 5867-5871.

[17] X. Wang, L. Yu, B. Y. Guan, S. Song, X. W. Lou, *Adv. Mater.* **2018**, *30*, 1801211.

[18] J. S. Kim, I. Park, E.-S. Jeong, K. Jin, W. M. Seong, G. Yoon, H. Kim, B. Kim, K. T. Nam, K. Kang, *Adv. Mater.* **2017**, *29*, 1606893.

[19] Y. Zheng, Y. Jiao, Y. Zhu, Q. Cai, A. Vasileff, L. H. Li, Y. Han, Y. Chen, S.-Z. Qiao, *J. Am. Chem. Soc.* **2017**, *139*, 3336-3339.

[20] S. Li, C. Cheng, X. Zhao, J. Schmidt, A. Thomas, *Angew. Chem .Int. Ed.* **2018**, *57*, 1856-1862.
